# Sensor setpoints that ensure compliance with microbial water quality targets for membrane bioreactor and chlorination treatment in on-site water reuse systems

**DOI:** 10.1016/j.wroa.2022.100164

**Published:** 2022-12-27

**Authors:** Eva Reynaert, Flavia Gretener, Timothy R. Julian, Eberhard Morgenroth

**Affiliations:** a Eawag, Swiss Federal Institute of Aquatic Science and Technology, 8600 Dübendorf, Switzerland; b ETH Zürich, Institute of Environmental Engineering, 8093 Zürich, Switzerland; c Swiss Tropical and Public Health Institute, 4051 Basel, Switzerland; d University of Basel, 4055 Basel, Switzerland

**Keywords:** Online sensors, Water reuse, Chlorination, Virus removal, Bacterial regrowth, Risk-based monitoring

## Abstract

Widespread implementation of on-site water reuse systems is hindered by the limited ability to ensure the level of treatment and protection of human health during operation. In this study, we tested the ability of five commercially available online sensors (free chlorine (FC), oxidation-reduction potential (ORP), pH, turbidity, UV absorbance at 254 nm) to predict the microbial water quality in membrane bioreactors followed by chlorination using logistic regression-based and mechanism-based models. The microbial water quality was assessed in terms of removal of enteric bacteria from the wastewater, removal of enteric viruses, and regrowth of bacteria in the treated water. We found that FC and ORP alone could predict the microbial water quality well, with ORP-based models generally performing better. We further observed that prediction accuracy did not increase when data from multiple sensors were integrated. We propose a methodology to link online sensor measurements to risk-based water quality targets, providing operation setpoints protective of human health for specific combinations of wastewaters and reuse applications. For instance, we recommend a minimum ORP of 705 mV to ensure a virus log-removal of 5, and an ORP of 765 mV for a log-removal of 6. These setpoints were selected to ensure that the percentage of events where the water is predicted to meet the quality target but it does not remains below 5%. Such a systematic approach to set sensor setpoints could be used in the development of water reuse guidelines and regulations that aim to cover a range of reuse applications with differential risks to human health.

## Highlights

• On-site water reuse improves water access, but user health must always be ensured.
• We predicted microbial water quality in MBR+chlorine systems using five sensors.
• ORP and free chlorine were closely linked to virus removal and bacterial regrowth.
• We propose systematic approaches to link microbial water quality to sensor targets.
• Such approaches can inform the development of guidelines and regulations.

## Graphical abstract

**Figure:**
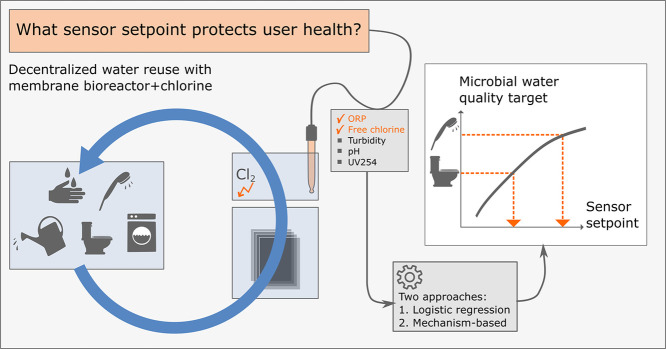

## 1 Introduction

On-site water reuse systems can offer flexible solutions to provide water and protect freshwater resources in water-scarce areas. The challenge for such systems is that microbial safety must be ensured at all times. Today, membrane bioreactors (MBRs) are increasingly used as a barrier in on-site water reuse systems – especially in small-scale systems with fewer than 50 people equivalent – and are considered a best available technology (Branch et al., 2016; Lesjean et al., 2011). Disinfection of the water is mostly achieved through chlorination, due to its broad-spectrum efficacy and low cost (Ikehata et al., 2018).

The high quality of water treated with MBR-based systems has multiplied the applications of reclaimed water beyond traditional reuse in agriculture (Angelakis et al., 2018; Chen et al., 2013), with novel technologies that allow to reuse water for on-site residential uses such as toilet flushing (Bair et al., 2015), showering (Gassie and Engelhardt, 2017), or hand washing (Reynaert et al., 2020). In parallel, the development of quantitative microbial risk assessment (QMRA) frameworks for water reuse has allowed to quantify the human health risk associated with water reuse and to propose systematic approaches to set microbial treatment targets that will keep the human health risk below a certain benchmark (Zhiteneva et al., 2020). Differential risks to human health make the definition of treatment targets for water reuse more complex than for drinking water applications, as the targets depend on the composition of the wastewater, the assumed volume of water ingested for a certain reuse application, and the pathogen considered. For instance, treatment targets will be more stringent for reuse for showering (assumed high volume of water ingested) compared to reuse for toilet flushing (assumed low volume of water ingested).

To regulate the quality of the reclaimed water, many countries have issued legal and regulatory frameworks for water reuse (water reuse frameworks, WRFs) such as guidelines, standards, regulations, and codes of practice (Reynaert et al., 2021). Many of these WRFs integrate a fit-for-purpose approach, defining different water quality classes depending on the reuse application, with different requirements in terms of treatment technology, permissible contaminant concentrations, and monitoring. To date, most WRFs require regular monitoring of fecal indicator bacteria to verify microbial water quality. As examples, the US EPA Guidelines for Water Reuse recommend daily monitoring of fecal coliforms and the Spanish Regulations for Water Reuse bi-weekly monitoring of *E. coli* (Ministry of the Presidency Spain, 2007; US EPA, 2012). Frequent monitoring of (only) fecal indicator bacteria as suggested in WRFs is problematic for two reasons:

1. In on-site systems, laboratory-based methods may no longer be suitable due to economic and organizational constraints and will results in delayed action due to low-frequency monitoring (Reynaert et al., 2021).
2. Fecal indicator bacteria do not provide information on virus removal and regrowth of bacteria in the treated water (Baggi et al., 2001), especially for MBR treatment, where bacteria are retained by the membrane but viruses may pass.

Finding adequate solutions for the monitoring of the microbial water quality would solve a major bottleneck for the widespread implementation of on-site water reuse technologies (Zhang et al., 2020). Online sensors and remote monitoring offer an opportunity for the management of on-site water reuse technologies to ensure treatment performance and protect public health. However, most WRFs do not provide quality targets for parameters indicative of microbial water quality that can be measured online. In light of increasingly diverse reuse applications, there is a need for flexible approaches to link sensor target stringency directly with differential risks to human health.

Online monitoring and control systems based on the oxidation-reduction potential (ORP) or free chlorine (FC) exist for various applications, and have been implemented for wastewater effluent chlorination (WERF, 2005) or swimming pool disinfection (Steininger, 1990) for decades. The current work expands this concept for online monitoring and control strategies for on-site water reuse systems using membrane bioreactor an chlorination treatment (MBR+chlorine).

We investigated the relationships between five commercially available and widely used online sensors – FC, ORP, pH, turbidity, and UV absorbance at 254 nm (UV254) – and the water quality using statistical and mechanism-based approaches. The microbial water quality is assessed in terms of (i) removal of enteric bacteria from the wastewater, (ii) removal of enteric viruses, and (iii) bacterial regrowth in the treated water.

## 2 Materials and methods

### 2.1 MBR+chlorine system

**Fig. 1:**
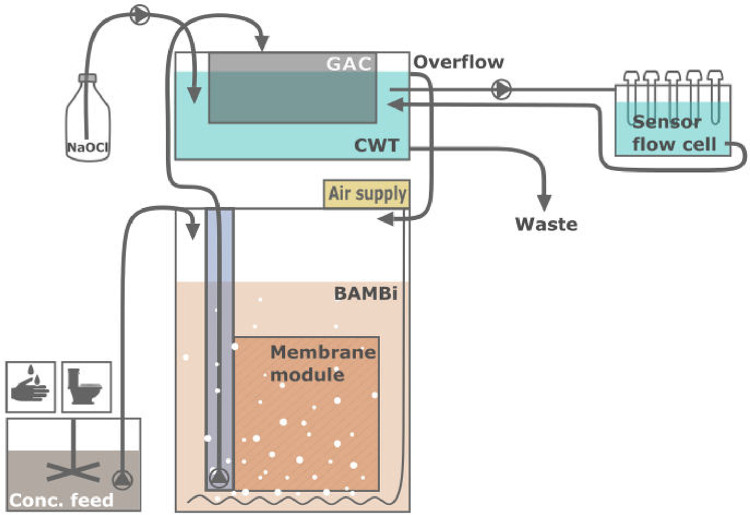
Process schematic for the biologically activated membrane bioreactor (BAMBi) configured with granular activated carbon (GAC) and chlorination post-treatment with a concentrated NaOCl solution. The clean water tank (CWT) is positioned above the BAMBi, so that the overflow water from the CWT can flow into the BAMBi. Water from the CWT is constantly recirculated through a flow cell containing a range of commercially available online sensors. In this laboratory setup, concentrated feed (representing handwashing or source-separated toilet flush water) is added to the BAMBi, with the same amount of water being removed from the CWT (waste).

The MBR+chlorine system used in this study is referred to as Water Wall (Reynaert et al., 2020, www.autarky.ch). The Water Wall consists of two main components: the core treatment takes place in a biologically activated membrane bioreactor (BAMBi, Künzle et al., 2015), after which the water is further treated and disinfected using granular activated carbon (GAC) and chlorine in a clean water tank (CWT, Fig. 1).

The BAMBi contained a standing sandwich membrane module (Microclear MCXL, Newterra, Langgöns, Germany) featuring a 150 kDa polyethersulfone ultrafiltration membrane (Microdyn-Nadir, Wiesbaden, Germany). Aeration was provided directly below the membrane module at a rate of 5 L/min. The BAMBi was operated in a gravity-driven membrane (GDM) configuration, where the transmembrane pressure is due to the water head (Peter-Varbanets et al., 2010). Water that passed through the membrane was collected in a permeate reservoir (10 cm polyvinyl chloride pipe connected to the membrane module permeate outlet, holding volume of 4 L), from where it was pumped through a GAC filter (Norit 830, ∼1.5 mm grain diameter, Cabot, Boston, USA) to the CWT every 13 min. In the CWT, a concentrated NaOCl solution (1’750 mg Cl_2_/L) was pumped into the tank at regular intervals. The pumping intervals were varied during the experiments to achieve a range of free chlorine concentrations in the CWT (0-2 mg/L). Mixing in the CWT was ensured through a submersed pump that was turned on for 30 s every 5 min. The tank volumes were 60 L water for the BAMBi and 25 L for the CWT, with an average hydraulic residence time (HRT) of 5 h in the CWT.

To study the effect of treating different types of water input, two Water Wall systems were operated in parallel in this testing: one with controlled feeding of handwashing greywater, and the other with toilet flush water (separated from the major part of urine and feces). The compositions of the 20x concentrated feed solutions are presented in SI 1 (Supplementary Information). For each reactor, a total of 3.75 L/day of concentrated feed was pumped into the BAMBi in a series of 50 feedings, evenly distributed throughout the day. This daily feed represents the loading that would be introduced to a total of 75 L water of real hand washing or source-separated toilet flush water. The same amount of water was removed from the system to maintain a constant volume. Other than changes in the chlorination (pumping intervals of the chlorine dosing pump to achieve a range of microbial water qualities), both Water Walls were operated under stable conditions throughout the testing.

### 2.2 Physicochemical water quality

#### 2.2.1 Online monitoring

Water from the CWT was constantly recirculated through flow cells equipped with a range of online sensors (flow rate 0.5 L/min, flow cell volume 0.25 L, corresponding HRT 0.5 min). Flow cells were used to house the online sensors because of the limited space in the CWT and to improve access and control of sensors. Selection criteria for these sensors were (i) commercial availability, with (ii) appropriate costs and dimensions for implementation in on-site applications, and (iii) expected mechanistic relationships between the sensor measurements and microbial water quality. Based on these criteria, five sensors were selected: ORP, FC, pH, turbidity and UV254 (Table 1). The proposed approaches to set sensor target values are, however, generalizable to novel sensors that may be more widely available in the future (e.g., fluorescence sensors).

**Table 1:** Specifications and expected link to microbial water quality of the sensors installed in the MBR+chlorine systems. E+H: Endress+Hauser, Reinach, Switzerland.

**Measurement**	**Sensor specification**	**Measurement principle**	**Expected link to microbial water quality**
Free chlorine (FC)	Digital free chlorine sensor Memosens CCS51D, E+H	Closed, membrane-covered measuring cell; reduction of free chlorine at the cathode	Free chlorine is the most effective chlorine form in disinfection with sodium hypochlorite (Kim and Hensley, 1997)
Oxidation-reduction potential (ORP)	Ceragel CPS72D, E+H	Ceramic diaphragm double chamber and double gel reference Platinum-ring	Measurement of the oxidizing or reducing tendency of the water, for chlorinated systems: oxidative capacity of all chlorine species (James et al., 2004)
pH	Orbisint CPS11D, E+H	Gel compact electrode with PTFE ring-diaphragm	Information on speciation of free chlorine (i.e., disinfection potential of the free chlorine) (Cherney et al., 2006)
Turbidity	Turbimax CUS52D, E+H	Nephelometric turbidity sensor (90° scattering) according to ISO7027	Turbidity can be linked to the chlorine demand of the water (LeChevallier et al., 1981) and to bacteria concentrations (Hess et al., 2021)
UV254 (only in system treating toilet flush water)	spectro::lyser, s::can, Vienna, Austria	UV absorbance at 254 nm (in principle: full UV-VIS spectra, but used as a proxy for a lower-cost sensor)	UV absorbance at 254 nm is a proxy for organic matter in the water and can correlate with bacteria and virus concentrations(Van Den Broeke et al., 2006)

#### 2.2.2 Offline monitoring

Samples for dissolved organic carbon (DOC), nitrite, nitrate and ammonium measurements were filtered at 0.45 μm (Nanocolor Chromafil membrane filter GF/PET 0.45 μm, Macherey Nagel, Düren, Germany) for sample conservation and stored at 4°C before the chemical analysis. DOC was measured using a total organic carbon analyzer (Shimadzu TOC-L, Kyoto, Japan). Ammonium was measured by gas-diffusion flow injection (Foss, Hillerød, Demark). Nitrite and nitrate were measured by means of ion chromatography (Metrohm 881, Herisau, Switzerland).

Free and total chlorine were measured immediately after sampling using a portable spectrophotometer (DR 1900, Hach, Loveland, USA) with corresponding test kits (DPD, 0–2 mg/L free chlorine, Hach, Loveland, USA).

### 2.3 Microbial water quality

Microbial water quality samples were taken under stable reactor operation, with constant chlorine concentrations for at least three HRT (∼15 h) in the CWT.

*E. coli* was used as an indicator for the removal of enteric bacteria from the wastewater. Samples from the concentrated feed and from the CWT were analyzed following the US EPA method 1603 (*E. coli* in water by membrane filtration). *E. coli* was used as an indicator for the removal of enteric bacteria from the wastewater, consistent with the use of *E. coli* or other fecal bacteria indicators (fecal coliform, total coliform, enterococci) within WRFs (e.g., US EPA, 2012). In short, the water samples were filtered through a membrane (S-Pak-Filter, 0.45 µm, Millipore Sigma, Burlington, USA) that was placed on m-TEC ChromoSelect agar (Millipore Sigma, Burlington, USA) and incubated at 44°C for 24 hours. The colony-forming units (CFU) on the plates were then enumerated. For the CWT, 100 mL of undiluted sample was analyzed, while the concentrated wastewater needed prior dilution (1:250’000 to 1:2’500’000) before analysis.

The bacteriophage MS2 was used as an indicator for the removal of enteric viruses from the wastewater. MS2 (ATCC 15597-B1) and its associated *E. coli* host (ATCC 700891) were purchased from the American Type Culture Collection (ATCC). MS2 stock solution was prepared by amplifying the initial MS2 stock solution in 1 L of *E. coli* culture. The double agar layer assay was used to enumerate infectious bacteriophages as plaque forming units (PFU) following the US EPA Method 1602. Briefly, 100 µL of *E. coli* host were mixed in soft agar (0.7% agar) and poured onto a hard agar plate (1.5% agar). Different from the described protocol, the MS2 sample was spotted onto the agar plate rather than mixing it with the soft agar, allowing to process multiple dilutions easily.

The overall log-removal value (LRV) of *E. coli* and MS2 was calculated according to Eq. (1):

(1) $$LRV_{overall}=-log_{10}\frac{N_{CWT}}{N_{feed}/f_{conc}}$$

where $N_{CWT}$ and $N_{feed}$ are the microbial indicator concentrations in the CWT and concentrated feed, respectively, and $f_{conc}$ is the concentration factor of the feed (20). When no indicators were detected in the CWT, the detection limits (1 CFU/100mL for E. coli, 10 PFU/mL for MS2) were used to calculate the LRVs. In this case, the reported $LRV_{overall}$ is reported as greater than the maximum detectable LRV.

The intact cell concentration (ICC) was used to indicate bacterial regrowth in the CWT. The ICC was determined with a flow cytometer (Cytoflex, Beckman Coulter, Brea, California, USA) using SYBR® Green I stain (ThermoFisher Scientific, Waltham, Massachusetts, USA) and propidium iodide (ThermoFisher Scientific, Waltham, Massachusetts, USA). Flow cytometry analysis was selected for bacterial quantification because of the simplicity and speed of processing large numbers of samples (relative to sequencing or qPCR techniques) and the ability to quantify bacteria that are not culturable using heterotrophic plate counts (Van Nevel et al., 2017). Flow cytometry has also been demonstrated to descriptively quantify changes in cell concentrations in system using chlorination or electrolysis as post-treatment (Ziemba et al., 2019). The detection limit of flow cytometry in this study was 29 cells/mL.

### 2.4 Calculation of sensor target values

Sensor target values were calculated for a range of log-removal targets (LRTs) for *E. coli* and MS2 and of allowable concentrations for ICC (upper and lower limits determined through the detection limits of the methods and concentrations of indicators in the feed), using a statistical approach and a mechanism-based approach.

#### 2.4.1 Logistic regression model to determine sensor target values

Logistic regression is a classification algorithm that predicts the probability p of a binary outcome (water quality meeting a certain target or not) based on one or more independent variables (sensor measurements). The binary outcome is the dependent variable, which is based on a binary classification of whether (1, safe) or not (0, unsafe) the acceptable microbial target is met for each observation within the dataset. The accuracy of prediction was calculated using leave-one-out cross validation. The leave-one-out cross validation procedure is appropriate for relatively small datasets, when an accurate estimate of model performance is required (Wong, 2015). The accuracy obtained with single sensors was compared with the best-performing combination of sensor measurements (except UV254) computed with the R package MuMIn (1.43.17) (Barton, 2022). UV254 was excluded, as there were significantly fewer data points available for this sensor (implemented only in the MBR+chlorine system treating toilet flush water). The error matrix presented in Table 2 was used to evaluate the models.

**Table 2:** Error matrix.

The false safe rate (FSR) and the false unsafe rate (FUR) were calculated as:

(2) $$FSR=\frac{FS}{FS+TU}$$

(3) $$FUR=\frac{FU}{FU+TS}$$

When computing logistic regression models based on only one sensor input (required for the calculation of sensor targets), the logistic regression equation has two parameters, α and β:

(4) $$p=\frac{1}{1+e^{-\left(\alpha +\beta x\right)}}$$

The goodness of fit was evaluated using McFadden's Pseudo-R^2^ (Smith and McKenna, 2013). Bootstrapping (R package boot version 1.3-28, 100’000 iterations) was used to calculate the 90% confidence intervals. Sensor target values were set to meet a 95% percent probability (lower confidence interval) that the water quality meets the microbial targets, i.e., for p = 0.95,

(5) $$x_{target}=\frac{-ln\left(\frac{1}{0.95}-1\right)+\alpha }{\beta }$$

This approach implies that the allowed FSR (sensor measurement predicts that the water quality meets a certain target when it does not) is 5%.

#### 2.4.2 Mechanism-based sensor target values

Relevant processes for the determination of mechanism-based sensor target values were: (a) retention/inactivation of bacteria and viruses in the MBR, (b) inactivation of bacteria and viruses during chlorination in the CWT, and (c) net regrowth of bacteria in the CWT.

(a) The LRV for retention/inactivation in the MBR was calculated as
  (6) $$LRV_{MBR}=-log_{10}\left(\frac{N_{post-MBR}}{N_{feed}/f_{conc}}\right)$$

where $N_{post-MBR}$ refers to the microbial indicator concentration after passage through the MBR (theoretical concentration in CWT without disinfection and without regrowth), and $N_{feed}/f_{conc}$ corresponds to the microbial indicator concentration in the diluted feed (see above).

(b) The LRV for inactivation in the CWT was based on a Chick-Watson model for disinfection:
  (7) $$-log_{10}\left(\frac{N\left(t\right)}{N_{CWT-steady,w/oCl2}}\right)=kC^{n}t$$

where N(t) is the microbial indicator concentration after contact time t, $N_{CWT-steady,w/oCl2}$ is the steady-state microbial indicator concentration in the CWT without chlorination, k is a reaction constant, C represents the disinfectant concentration/capacity, and n is the coefficient of dilution (a fitting parameter).

Chick-Watson models have mainly been used with the chlorine concentration as a measure of disinfection capacity (Peleg, 2021). In this study, we also included the ORP as a measurement of disinfection capacity. This needed an expansion of the Chick-Watson model for measures of the disinfection capacity that are not zero without disinfection, accounting for a baseline measurement of disinfection capacity:

(8) $$-log_{10}\left(\frac{N\left(t\right)}{N_{CWT-steady,w/oCl2}}\right)=k{\left(C-C_{baseline}\right)}^{n}t$$

where $C_{baseline}$ is the disinfectant capacity without chlorination.

Chick-Watson models have been used to model the dynamics of disinfection over time. Testing in our MBR+chlorine systems showed that the concentrations of microbial indicators stabilize for long contact times (results in SI 2). This is also in accordance with a study from Cheswick et al. (2020), which shows that the ICC stabilizes at different values for different chlorine concentrations and from Hornstra et al. (2011), which shows strong tailing for MS2 disinfection at low concentrations of chlorine dioxide.

The Chick-Watson model was adapted to account for this time-independent disinfection, assuming that after a time $t_{steady}$ << HRT, microbial indicator concentrations stabilize and we reach the maximum LRV due to disinfection.

(9) $$LRV_{disinfection}=-log_{10}\left(\frac{N_{CWT-steady}}{N_{CWT-steady,w/oCl2}}\right)=k^{\prime }{\left(C-C_{baseline}\right)}^{n}$$

where $N_{CWT-steady}$ represents the microbial indicator after time $t_{steady}$ (i.e., steady-state concentration in CWT) and $k^{\prime }=kt_{steady}$.

The conditions for using Eq. (9) are that $N_{CWT-steady}$ > 0 and $C>C_{baseline}$ (otherwise $LRV_{disinfection}$= 0).

(c) The net regrowth at steady state can be calculated as (condition: $N_{post-MBR}$ > 0):
  (10) $$RG_{steady}=log_{10}\left(N_{CWT-steady,w/oCl2}/N_{post-MBR}\right)$$

where $RG_{steady}$ is the (net) regrowth at steady-state.

Combining the three mechanism-based model parts, the final equations are:

(11) $$LRV_{overall}=LRV_{MBR}+LRV_{disinfection}-RG_{steady}$$

(12) $$N_{CWT-steady}=N_{CWT-steady,w/oCl2}-N_{CWT-disinfected}$$

$N_{CWT-disinfected}$ represents the concentrations of indicator organisms inactivated by disinfection, which we can rewrite as (using Eq. (9)):

(13) $$N_{CWT-disinfected}=N_{CWT-steady,w/oCl2}-N_{CWT-steady}=N_{CWT-steady,w/oCl2}-10^{LRV_{disinfection}}\cdot N_{CWT-steady,w/oCl2}$$

Inserting Eq. (13) into Eq. (12) and taking the logarithm, we can rewrite the steady-state concentration as:

(14) $$log_{10}\left(N_{CWT-steady}\right)=log_{10}\left(N_{CWT-steady,w/oCl2}\right)-LRV_{disinfection}$$

These equations can further be simplified for the specific microbial indicators:

Removal of *E. coli*:

$N_{CWT-steady}$ and $N_{CWT-steady,w/oCl2}$<1 CFU/100 mL, due to complete retention in the MBR (see Results section 3.2), so there is no regrowth and removal through disinfection in the CWT.

(15) $$LRV_{overall,E.coli}=LRV_{MBR}=-log_{10}\left(\frac{1}{N_{feed}/f_{conc}}\right)$$

Removal of MS2:

Growth of MS2 is assumed to be nonexistent or negligible, given the absence of detectable *E. coli* (as the required MS2 host) in the CWT. We can thus replace $N_{post-MBR}$ (not measured) with $N_{CWT-steady,w/oCl2}$ (measured):

(16) $$LRV_{overall,MS2}=LRV_{MBR}+LRV_{disinfection}=-log_{10}\left(\frac{N_{CWT-steady,w/oCl2}}{N_{feed}/f_{conc}}\right)+k^{\prime }{\left(C-C_{baseline}\right)}^{n}$$

Regrowth of ICC:

(17) $$log_{10}\left(N_{CWT-steady}\right)=log_{10}\left(N_{CWT-steady,w/oCl2}\right)-k^{\prime }{\left(C-C_{baseline}\right)}^{n}$$

The parameters $C_{baseline}$ and $N_{CWT-steady,w/oCl2}$were calculated as the average values of the respective measurements when the systems were operated without chlorination. The constants $k^{\prime }$ and n were chosen such as to optimize the fit of $\left(C-C_{baseline}\right)$ vs $LRV_{disinfection}$ using the function *nls* (nonlinear least squares) from the R package stats (version 4.1.0). Assuming a normal distribution of the errors, the 90% confidence intervals were calculated by multiplying the standard deviation of the fit with a z-score of 1.65.

## 3 Results

### 3.1 Reactor operation

The operation of the MBR+chlorine system treating source-separated toilet flush water was stable over the four months of experiments, with DOC concentrations consistently below 3 mg_C_/L and ammonium mostly below the limit of detection of 0.02 mg_N_/L in the permeate (see SI 3). In contrast, DOC and ammonium concentrations in the MBR+chlorine system treating handwashing water were more variable, with increased concentrations (up to 13 mg_C_/L DOC and 27 mg_N_/L ammonium) during the first weeks of operation, and another peak towards the end of the experiments. During these periods, there was no FC in the treated water, however, there were varying concentrations of combined chlorine. The pH measurements ranged between 6.8 and 7.7 for the system treating toilet flush water and between 7.0 and 7.7 for the handwashing water system, implying that the speciation of the hypochlorous acid (pK_a_ = 7.5) varied during the experiments. The full dataset collected for this study can be found under doi.org/10.25678/0007NQ.

### 3.2 Relationships between sensor measurements and microbial indicators

**Fig. 2:**
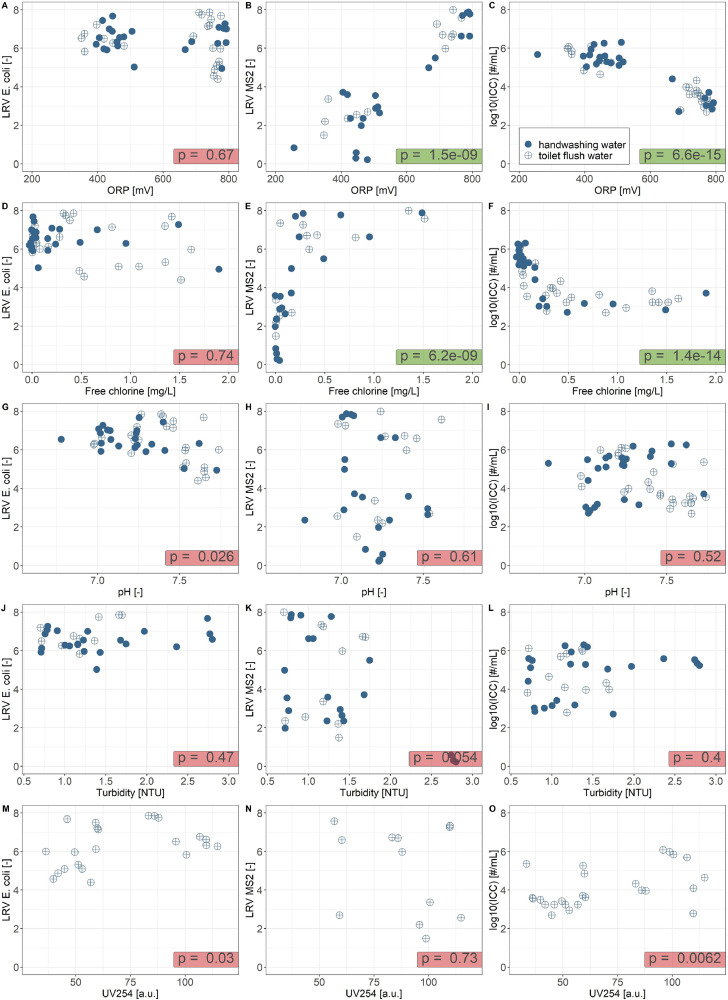
Log-removal values (LRV) for *E. coli*, LRV for MS2 and log_10_-value of ICC as a function of the oxidation-reduction potential (OPR), free chlorine, pH, turbidity and UV absorbance at 254 nm (UV254, with arbitrary units a.u.). p-value: significance of Spearman's rank correlation (p ≤ 0.001 is considered significant and highlighted in green). For LRV *E. coli*, all values are maximum detectable LRVs (no *E. coli* detected post-membrane).

Fig. 2 presents the fifteen relationships (five sensors vs. three microbial indicators) that were investigated. As there were limited systematic differences between the datasets (p-value of t-test > 0.05 for all parameters, except of pH, where p = 0.0038), the further data analysis was done on the combined dataset. Cross-correlation plots showing the relationship between all parameters are presented in SI 4.

*E. coli* was not detected post-membrane. This means that the maximum detectable LRV was determined by the concentration of *E. coli* in the concentrated feed and the method's limit of detection and was thus not related to any of the sensor measurements in the treated water. Sensor measurements that were significantly correlated with LRV MS2 and log_10_ICC (Spearman's rank correlation, p ≤ 0.001) included ORP and FC. In contrast, the pH value, turbidity and UV254 were not significantly correlated with any of the microbial indicators.

### 3.3 Logistic regression-based sensor target values

For a range of microbial water quality targets, the dataset was classified into the binary categories 0 (water quality does not meet microbial target; unsafe) and 1 (water quality meets microbial target; safe). Logistic regression equations were computed for each microbial target. For MS2, logistic regressions were computed for LRVs between 4 and 6.5. For ICC, logistic regressions were computed for log_10_-concentrations between 3.5 and 6 (see Table 3 for selection of relevant ranges). Table 3 presents the accuracy of prediction (percentage of correct predictions out of all predictions) for these logistic regression models based on only ORP or FC (significantly correlated with microbial water quality) compared to the best-performing combination of sensors using leave-one-out cross-validation. With one exception (log_10_ ICC ≤ 3.5), the largest difference between the better-performing single-sensor model and the best-performing combination of sensors is 4%. However, it was not always the same sensor, ORP or FC, which had the higher accuracy. ORP and FC alone can thus predict the microbial water quality nearly as well as the combination of all sensors.

**Table 3:** The accuracy of prediction (using leave-one-out cross-validation) of logistic regression models based on single sensors (ORP: oxidation reduction potential and FC: free chlorine) compared to the best-performing combination of sensors (without UV254). LRT: log-removal target. ICC: intact cell concentration. Cond: conductivity. Turb: turbidity.

	ORP	FC	Best model	Sensors included in best model
**LRT MS2**				
≥ 3.5	LRV MS2 can be up to 3.5 without disinfection			
≥ 4	100%	74%	100%	ORP + pH
≥ 4.5	100%	74%	100%	ORP + pH
≥ 5	82%	93%	93%	FC
≥ 5.5	74%	72%	74%	ORP
≥ 6	73%	65%	74%	ORP + Cond + pH
≥ 6.5	73%	67%	74%	ORP + Cond + pH
≥ 7	Sensor target value is above maximum measurements			
**log_10_ ICC**				
≤ 3	close to the limit of detection for ICC			
≤ 3.5	69%	61%	100%	ORP + pH
≤ 4	76%	74%	77%	ORP + FC
≤ 4.5	100%	76%	100%	ORP + pH
≤ 5	81%	75%	81%	ORP
≤ 5.5	63%	66%	70%	FC + pH + Turb
≤ 6	64%	64%	65%	pH
≤ 6.5	max log_10_ICC is 6.3			

**Fig. 3:**
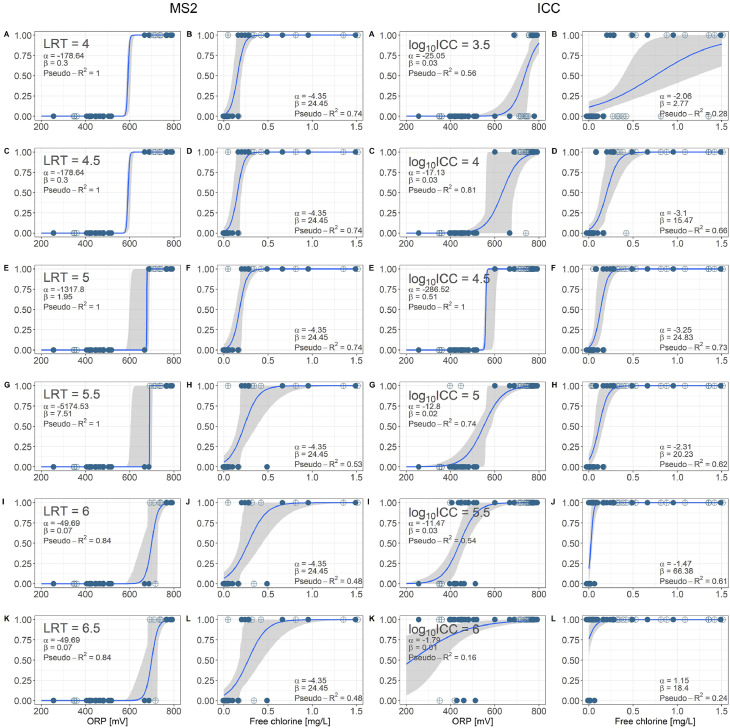
Logistic regression models for LRV of MS2 and log_10_-value of ICC as a function of the oxidation-reduction potential (ORP) and free chlorine concentrations. LRT: log-removal target for MS2. 0 = microbial indicator does not meet the microbial water quality target, 1 = meets target. α and β are parameters for the logistic equation, while McFadden's Pseudo-R^2^ evaluates the goodness of fit. The grey areas represent bootstrapped 90% confidence intervals. Blue filled circle: reactor treating handwashing water. Blue crossed circle: reactor treating source-separated toilet flush water.

Fig. 3 presents the logistic regression models for the four combinations of sensor measurements and microbial indicators that are significantly correlated. The pseudo-R^2^ is above 0.4 for all but one microbial target (log_10_ICC = 6), indicating a very good fit according to Louviere et al. (2000). For some regressions, the sensor measurements could be divided into two perfectly separated clusters, resulting in a pseudo-R^2^ of 1 (perfect fit). A perfect fit implies that the error on the model can no longer be directly calculated due to a lack of data in the transition range. Bootstrapping was thus used to calculate the 90% confidence intervals. As can be seen in Fig. 3, bootstrapping produces relatively narrow confidence intervals for perfect separation, due to the limited data resolution in the transition range.

For regressions with a good fit (Pseudo-R^2^ > 0.4), sensor target values for ORP and FC were set to meet a 95% percent probability that the water quality meets the microbial targets (using the lower bound of the confidence interval). The sensor target values for ORP and FC are presented in Table 4(A). The selected 95% probability reduces the FSR (i.e., model predicts safe water when it is not) to a maximum of 5% for the entire data set. However, this comes at the cost of an elevated FUR (up to 40%), indicating that in some cases, the microbial targets are met at lower sensor values than the set sensor target. While the recommended sensor targets are consistent for the LRT of MS2 (increasing for increasingly stringent quality targets), some targets could not be calculated for ICC (recommended target values exceeding maximum measurements; fit of regression too poor). One value stands out for ICC: the ORP target of 605 mV (range 600-620 mV) for a log_10_ICC ≤ 4.5 is lower than the recommendation for a log_10_ICC ≤ 5 (690 mV, 605-720 mV), however, the ranges of ORP targets overlap. This inconsistency is due to the perfect separation of the data set for the log_10_ICC ≤ 4.5, where bootstrapping likely underestimates the 90% confidence interval.

**Table 4:** Oxidation-reduction potential (ORP) and free chlorine sensor target values with 90% confidence interval for a range of microbial targets for LRV MS2 and log_10_-value of ICC, based on logistic regression models (A) or mechanism-based models (B). ORP: rounded to next 5 mV. FC: rounded to next 0.05 mg/L. ACC: overall accuracy of prediction. FSR: false safe rate (model predicts safe water when water is not safe). FSR was defined to be ≤ 5% for the logistic regression model. FUR: false unsafe rate (model predicts unsafe water when water is safe). ACC, FSR and FUR all reported for the upper bound of the 90% confidence intervals. Bold: recommended sensor target values.

		**A. Logistic regression model**									**B. Mechanism-based model**							
		**ORP [mV]**				**Free chlorine [mg/L]**					**ORP [mV]**				**Free chlorine [mg/L]**			
		Target	ACC	FSR	FUR	Target	ACC	FSR	FUR		Target	ACC	FSR	FUR	Target	ACC	FSR	FUR
**LRT MS2**																		
≥ 3.5		LRV MS2 can be up to 3.5 without disinfection									LRV MS2 can be up to 3.5 without disinfection							
≥ 4		605 (600-620)	100%	0%	0%	0.3 (0.15-0.35)	77%	0%	23%		580 (510-**645**)	100%	0%	0%	0.07 (0.02-**0.6**)	71%	0%	29%
≥ 4.5		605 (600-620)	100%	0%	0%	0.3 (0.15-0.35)	77%	0%	23%		605 (540-**675**)	97%	0%	3%	0.11 (0.03-**0.81**)	69%	0%	31%
≥ 5		680 (610-695)	94%	0%	6%	0.3 (0.2-0.4)	80%	0%	20%		635 (565-**705**)	94%	0%	6%	0.17 (0.03-**1.08**)	65%	0%	35%
≥ 5.5		690 (620-705)	97%	0%	3%	0.5 (0.2-0.85)	71%	0%	29%		665 (595-**735**)	91%	0%	9%	0.26 (0.03-**1.4**)	66%	0%	34%
≥ 6		745 (685-765)	85%	0%	15%	0.6 (0.25-0.95)	71%	0%	29%		695 (625-**765**)	85%	0%	15%	0.38 (0.04-**1.78**)	63%	0%	37%
≥ 6.5		745 (685-765)	85%	0%	15%	0.6 (0.25-0.95)	71%	0%	29%		720 (650-**790**)	70%	0%	30%	Sensor target values > max sensor measurements			
≥ 7		Sensor target value > max sensor measurements									Sensor target value is above maximum measurements							
**log_10_ ICC**																		
≤ 3		Close to the limit of detection for ICC									Close to the limit of detection for ICC							
≤ 3.5		Sensor target values > max sensor measurements									Sensor target values > max sensor measurements							
≤ 4		745 (615-780)	60%	0%	40%	Pseudo-R^2^ < 0.4					685 (585-**775**)	69%	0%	31%	0.28 (0.03-**1.86**)	57%	0%	43%
≤ 4.5		605 (600-620)	100%	0%	0%	0.26 (0.13-0.32)	83%	0%	17%		625 (500-**715**)	92%	0%	8%	0.08 (0.02-**0.91**)	66%	0%	34%
≤ 5		690 (605-720)	85%	0%	15%	0.27 (0.13-0.35)	79%	0%	21%		545 (450-**655**)	96%	0%	4%	0.03 (0.02-**0.40**)	77%	0%	23%
≤ 5.5		560 (480-630)	77%	0%	23%	0.07 (0.03-0.13)	79%	0%	21%		450 (450-**585**)	77%	0%	23%	0.02 (0.02-**0.12**)	77%	0%	23%
≤ 6		Pseudo-R^2^ < 0.4									450 (450-**500**)	63%	2%	35%	0.02 (0.02-**0.04**)	72%	2%	26%
≤ 6.5		Max log_10_ICC is 6.3									Max log_10_ICC is 6.3							

### 3.4 Mechanism-based sensor target values

**Fig. 4:**
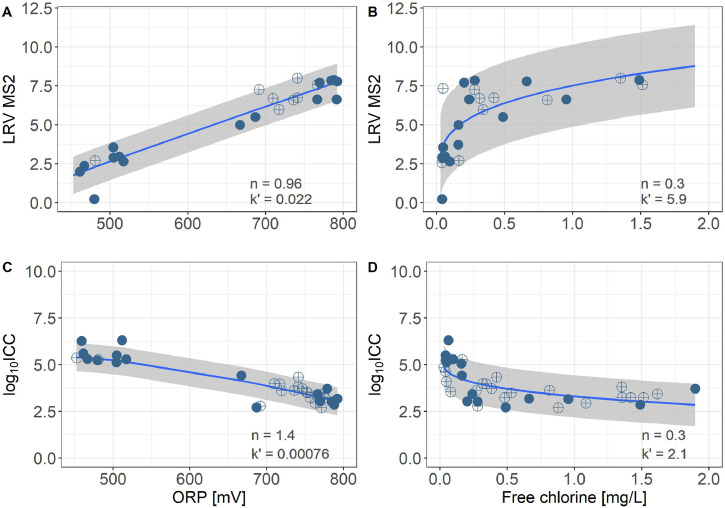
Mechanism-based models for LRV of MS2 and log_10_-value of ICC as a function of ORP and free chlorine concentrations for the disinfection part. Blue line: model prediction. Blue filled circle: reactor treating handwashing water. Blue crossed circle: reactor treating source-separated toilet flush water. Grey area: 90% confidence interval. Note: the disinfection model implicitly assumes that.

Note: the disinfection model implicitly assumes that

Fig. 4 presents the results for the mechanism-based disinfection models for MS2 and ICC. For *E. coli*, the model would predict a constant removal independent of the sensor measurements, as *E. coli* was consistently removed by the MBR. When using ORP as a predictor for the microbial indicators, the disinfection model predicts a constant LRV of 1.7 for MS2, respectively a constant concentration of intact cells of 5.4-log up to the ORP baseline value of 450 mV. Above this baseline value, there is an almost linear relationship between ORP and both microbial indicators, as the optimal dilution coefficient n in Eq. (9) is close to 1. When using FC as a predictor, the disinfection model predicts a constant LRV of 1.7 for MS2, respectively a constant concentration of intact cells of 5.4-log up to the FC_baseline_ value of 0.02 mg/L. Above this baseline value, the microbial indicators are related to (FC-FC_baseline_)^0.3^, where 0.3 is the optimal dilution coefficient. The mechanism-based sensor target values (with 90% confidence intervals) are presented in Table 4(B).

(18) $${\left(ORP-ORP_{baseline}\right)}^{n_{ORP}}=K{\left(FC-FC_{baseline}\right)}^{n_{FC}}$$

where $K=k_{ORP}^{\prime }$/$k_{FC}^{\prime }$. This assumption is discussed in SI 5.

### 3.5 Comparison of recommended sensor target values

The sensor target values Table 4 were set in a way to minimize the FSR (situations where the sensor value would predict safe water when the water is not safe) to 5%. This comes at the cost of overall accuracy, as there are many events where the water is safe even at lower sensor values.

Sensor targets for ORP predicting LRV MS2 are generally consistent between the logistic regression models and the mechanism-based model, with somewhat higher ORP targets for the mechanism-based models, which are thus recommended as target values (bold values in Table 4). In contrast, there are some differences between ORP targets predicting log_10_ICC. This is mainly due to the inconsistencies of the logistic regression models due to perfect separation of the dataset (see Section 3.3). For this reason, the mechanism-based sensor target values are recommended.

FC sensor target values using the mechanism-based models are almost twice as high as those from the logistic regression models (for both LRV MS2 and log_10_ICC). These higher targets for the mechanism-based model can be attributed to the high standard deviation of the FC measurements, leading to wide confidence intervals for the mechanism-based model. Here, the more conservative target values from the mechanism-based models are selected, too, as they better reflect the variation of FC measurements at low FC values. For the logistic regression-based models, there is a loss of information on the variability of the microbial water quality due to the binary classification.

## 4 Discussion

### 4.1 Interpretation of *E. coli* (removal of enteric bacteria) vs. MS2 (removal of enteric viruses) vs. ICC (regrowth)

In the tested MBR+chlorine systems, *E. coli* was consistently removed by the MBR. The quantifiable LRV was thus limited by the concentration in the feed, and the reported LRV has to be interpreted as a maximum-detectable LRV. This result was expected, as the nominal membrane pore size (∼0.04 µm) is more than one order of magnitude smaller than the size of *E. coli* (∼1 µm). While *E. coli* (or other enteric bacteria indicators) can be used to verify membrane integrity, it cannot be used to evaluate the overall performance of a functioning MBR+chlorine system inclusive other microbial threats including virus. Therefore, *E. coli* is not useful as the sole indicator for the determination of sensor target values.

Unlike *E. coli*, the enteric virus indicator MS2 concentrations in the CWT varied as a function of the operation of the MBR+chlorine systems. The calculated LRVs are thus meaningful and can be compared with LRTs determined through QMRA. For water reuse systems, such LRTs are usually calculated for specific combinations between a water source (e.g., greywater) and a reuse application (e.g., toilet flushing). The presented relationships between sensor measurements and LRVs can then be used to select a sensor target value for specific combinations of wastewater sources treated in a MBR+chlorine system and aiming to produce water for a certain reuse application.

In contrast, it is not possible to set concentration targets for ICC based on QMRA, as the ICC does not necessarily indicate presence or concentration of pathogens, and is thus not directly linked to microbial risk. However, several studies have suggested that ICC can be used to evaluate process performance (Cheswick et al., 2019; Van Nevel et al., 2017). While the ICC may vary between water reuse systems, there should not be abnormal changes in ICC in the same system during normal operation. We can thus define sensor target values that will ensure that the typical ICC (for a specific system) is not surpassed.

### 4.2 Best-performing sensors to predict the microbial water quality

Our results show that ORP and FC are closely linked to the microbial water quality, while turbidity and UV254 are not. Combining information from multiple sensors did not generally improve the prediction accuracy of logistic regression models.

#### 4.2.1 ORP and FC are closely linked to the microbial water quality

ORP and FC were the only two sensors that were significantly linked to the microbial water quality, with ORP-based models generally performing better (perfect separation of the data for many logistic regression models, lower relative standard deviation for mechanism-based models). This closer relationship for ORP compared to FC is supported by three theoretical considerations:

1. FC is the sum of hypochlorous acid (HOCl) and hypochlorite (OCl^−^). The speciation between these two compounds is determined by the pKa (∼7.5) and is thus pH-dependent. HOCl is the stronger disinfectant of the two species, with a specific lethality for viruses and bacteria that is around 100x higher than for OCl^−^ (Kim and Hensley, 1997). This pH-dependence of the disinfection capacity of the FC is accounted for in the ORP measurements, as OCl^−^ has a lower standard oxidation potential than HOCl (Kim and Hensley, 1997).
2. In the presence of ammonium, there is formation of chloramines, which also act as disinfectants, but are much less efficient than FC (Kim and Hensley, 1997). Measured free chlorine does not account for the disinfection by chloramines. In contrast, this increased disinfection capacity is accounted for in the ORP measurement, as the oxidation capacity of the chloramine is included in the overall ORP.
3. The response of ORP to chlorine is logarithmic (Nernst equation), which allows for an accurate detection of low chlorine concentrations. This advantage of ORP compared to FC is also visible in Fig. 3, where a complete separation of the microbial water quality indicators based on ORP is possible, but not based on FC, leading to a higher accuracy of prediction for models using ORP as an input to predict the microbial water quality.

The dataset used in this study captures the variability of the FC disinfection capacity due to changes in the pH and changes in chloramine concentrations, as the testing was done under variable pH concentrations (above and below the pKa) and included occurrence of ammonium in the treated water (see SI 3). The superiority of ORP as a measure of disinfection capacity has also been observed and commented in other laboratory studies (Kim and Hensley, 1997; Victorin et al., 1972).

For the practical implementation of ORP and FC sensors, two additional factors become important besides the strength of the link between sensor measurements and the microbial water quality: (1) most FC sensors cannot be implemented without an online measurement of the pH (to transform HOCl concentration into FC), which requires regular calibration, or are only applicable in a restricted pH range (e.g., 3-electrode sensors typically working for pH 6-9). (2) Non-chlorine based ORP may interfere with the ORP measurements. A study conducted by the Water Environment and Reuse Foundation sought to determine whether ORP or FC is better suited to control chlorine dosing in wastewater treatment plant effluents (WERF, 2005). The study concludes that for the chlorination of wastewater treatment plant effluents no technology was clearly superior for all criteria considered (provide information to meet effluent requirements; provide process control system reliability; minimize requirements; and minimize chemical use). However, ORP had a lower correlation with fecal coliforms in the chlorinated water. The main reason stated for the poorer performance of ORP were fluctuations of the ORP in the wastewater (between ∼200 mV and 500 mV), as a result of the weather conditions (dry vs. wet) and of industrial discharge. We expect the influent composition of decentralized water reuse systems to be less variable (no dependency on the weather, no industrial input), however, this needs confirmation from field campaigns.

#### 4.2.2 Turbidity and UV254 are not linked to the microbial water quality

Online monitoring of turbidity is recommended by several WRFs, and others have reported strong correlations between turbidity and the total cell concentration determined by flow cytometry (intact cells + damaged cells) (Hess et al., 2021). We assume that the lack of correlation between turbidity and ICC is due to the low ICC concentrations in the tested MBR+chlorine systems compared to concentrations reported by Hess et al. (2021) (∼1 order of magnitude higher) who studied an MBR system without chlorine disinfection.

Similarly, online monitoring of UV254 can be used as a proxy for organic matter concentrations (Van Den Broeke et al., 2006). However, the organic carbon contained in the ICC represents only a tiny fraction of the total DOC (< 0.01%, assuming 10^7^ cells = 1 µg DOC, Ziemba et al. (2020)). Natural variations of the DOC of the treated water thus by far exceeded changes in DOC caused by changes of the ICC. Direct correlations between pathogen contamination and UV-vis spectra have not been found to date (Carré et al., 2017).

Although we demonstrate lack of correlation of turbidity and UV254 with the microbial indicators in the tested MBR+chlorine system, these sensors can provide information on membrane integrity and may also hold promise for alternative treatment schemes (e.g., ozonation) (Katko and Højris, 2019).

### 4.3 Comparison of logistic regression and a mechanism-based models to define sensor targets

There is an ongoing debate whether data-driven or mechanistic models are better suited for the online-monitoring of critical process variables (Solle et al., 2017). The main advantages of logistic regression as a purely statistical approach lies in the low requirements of assumptions that need to be fulfilled and in the straightforward interpretation of results. Basic assumptions that must be met for logistic regression include only the independence of errors, linearity of independent variables and log odds, and the absence of multicollinearity (Stoltzfus, 2011). Key assumptions made for linear regression (linear relationship between dependent and independent variable, normal distribution of residuals and homoscedasticity) are not required. For the setting of risk-based sensor target values, where we want to limit the FSR, logistic regression allows to transparently select corresponding sensor values.

In this study, a major limitation of the logistic regression was the perfect separation of the water quality classes into two groups due to the small sample size and lack of data in the transition zones. Bootstrapping was used to estimate the confidence intervals. However, in the absence of prediction errors, these confidence intervals were relatively narrow. This led to an overconfident selection of sensor target values that may not capture the real variability in microbial water quality close to the recommended sensor target values. Bayesian analysis with non-informative prior assumption has been recommended for logistic regression with perfect separation (Gelman et al., 2008). However, the resulting confidence intervals are very wide due to the relatively small size of the dataset (see SI 6). Furthermore, it was not possible to collect data in the transition zone for ORP, as the ORP increased to high values as soon as there were detectable concentrations of FC in the water. If the perfect separation also holds at larger sample sizes, this would make ORP an excellent indicator of the microbial water quality and delineate a clear threshold for sensor targets.

The mechanism-based approach was a more adequate choice for the current dataset, as the dataset is relatively small, but we have knowledge about the main processes driving the microbial water quality (i.e., retention in the MBR, chlorine disinfection, regrowth in the stored water). While combining these processes follows a mechanistic approach, the modelling of the disinfection process itself (Chick-Watson) is phenomenological (data-derived). Such hybrid models, building on understanding of mechanisms in combination with data-driven methods, are increasingly used to model water reuse systems, as they combine the advantages of mechanistic (interpretability of results and extrapolation power) with those of data-driven models (e.g., learning unknown relationships) (Schneider et al., 2022).

### 4.4 Comparison with sensor target values from water reuse frameworks (WRFs)

An inspection of 19 WRFs (details presented in SI 7) shows that most WRFs define different water quality classes depending on the reuse application, setting stricter requirements for acceptable treatment technologies, permissible contaminant concentrations, and monitoring for higher-risk applications. Some WRFs explicitly link the reuse application to different LRTs, such as the Australian Guidelines for Water Recycling (NRMMC/EPHC/AHMC, 2006) and the Western Australian Code of Practice for the Reuse of Greywater (Department of Health Western Australia, 2010). However, the majority of WRFs set requirements in terms of final concentrations in the treated water, sometimes combined with higher monitoring frequencies for higher-risk applications.

In terms of chlorine concentration, the majority of WRFs set requirements for the total chlorine residual (sum of free and combined chlorine). Depending on the water composition (concentration of organics and ammonia), the disinfection capacity of the total chlorine is significantly lower than the disinfection capacity of the same concentration of free chlorine (Kim and Hensley, 1997). Many WRFs set a requirement of 1 mg/L for total chlorine, usually in combination with a minimum contact time (e.g., 15 min or 30 min). Assuming all chlorine was present as free chlorine, this would correspond to an expected LRV MS2 of ∼5 and log_10_ICC of ∼4.5 for the MBR+chlorine system in the current study (Table 4), which is relatively low for many of the reuse applications intended (e.g., toilet flushing). Using the average sensor targets from Table 4 (as opposed to the recommended 95% upper confidence level), we would predict > 6 LRV MS2 and < 4.5 log_10_ICC. However, these predictions do not account for the variability in microbial indicator removal/concentrations and sensor measurements, and still assume that the 1 mg/L of chlorine is present as FC. Overall, it seems that WRF requirements for total chlorine may not be sufficient to ensure adequate virus removal in MBR+chlorine systems designed for high-quality applications. Only the Californian requirements (concentration-time (CT) value of 450 mg·min/L with a contact time of at least 90 min, i.e., a chlorine concentration of around 5 mg/L), requiring a LRV MS2 of at least 5, are more conservative than the sensor target values proposed in this study (State Water Resources Control Board California, 2018). However, Hirani et al. (2014) reported that lower CT-values of 30 mg·min/L are sufficient to meet California's Title 22 disinfection requirements in MBR effluents.

Currently, no WRF sets requirements for the ORP of the treated water, but the Canadian Guidelines for Domestic Reclaimed Water for Use in Toilet and Urinal Flushing report ORP as a proxy to monitor the chlorine residual online (Health Canada, 2010). There are, however, various ORP requirements in guidelines for bathing waters. For instance, the WHO recommends an ORP of at least 720 mV, although it is suggested that appropriate values should be determined on a case by case basis, the German Environment Agency requires a minimum ORP of 750-770 mV depending on the pH, and the New South Wales Health Protection (Australia) requires an ORP of 720 mV (German Environment Agency, 2006; New South Wales Health Protection, 2013; WHO, 2006). These requirements are more protective of human health (corresponding to LRV MS2 of ∼5.5-6 and log_10_ICC of ∼5-4.5 in the current study) compared to the typical 1 mg/L (total) chlorine requirement for reclaimed water.

### 4.5 Applicability of sensor targets to other MBR+chlorine systems

The testing was performed with MBR+chlorine systems that treated two different types of wastewaters with similar relationships between sensor measurements and microbial indicators. This is an indication that the same phenomena may apply to MBR+chlorine systems treating a range of wastewater compositions. However, measurements from other MBR+chlorine systems will be required to validate the presented models and suggested sensor target values, as this study does not report model performance on a separate test dataset.

For MS2, the removal was due to retention in the MBR and inactivation through chlorine. For the retention of viruses in the MBR, a review on virus removal in full-scale submerged MBRs reports LRVs between 1.1 and 7.1 (O'Brien and Xagoraraki, 2020). The MS2 removal attributed to the MBR in this study (LRV of 1.7) is at the low end of the values reported in literature. For the disinfection, the two MBR+chlorine systems used in this testing achieved complete nitrification most of the time, with ammonia concentrations in the permeate below 0.2 mg_N_/L, resulting in low concentrations of chloramines. Overall, the proposed sensor targets for LRV MS2 are thus conservative, as the actual removal of viruses may be higher in other MBR+chlorine systems, due to higher removal in the MBR and the presence of chloramines during disinfection.

Regrowth can be due to growth of suspended organisms and to detachment of biofilm in the storage tank. The concentration of assimilable organic carbon (AOC), nutrients and the temperature have been shown to be important parameters determining regrowth of bacteria after ultrafiltration (Nguyen et al., 2017; Nocker et al., 2020). In this study, we used two different wastewaters, thus introducing some variability in the treated water composition (AOC, nutrients). In contrast, the temperature was relatively constant throughout the testing. If detachment of biofilm is a major contributor, the inclusion of a biological activated carbon filter and flow variability are additional relevant parameters that will determine the ICC in the treated water (Hess et al., 2021). We thus assume that it will be necessary to recalibrate the ICC models for MBR+chlorine systems that do not include an activated carbon filter.

Depending on the validation results using other MBR+chlorine systems, this study offers specific sensor target values for MBR+chlorine systems (likely for MS2) or approaches to set sensor target values for specific configurations of MBR+chlorine systems (likely for ICC).

## 5 Conclusion

• ORP and FC, which are both proxies for the efficacy of chlorination, are closely related to the microbial quality of reclaimed water treated with MBR+chlorine systems, while turbidity, pH and UV254 are not.
• We propose a mechanism-based methodology to set sensor target values that are linked to the microbial water quality in a transparent way.
• For ORP and FC, we recommend sensor target values for different microbial water quality targets (in terms of virus removal and bacterial regrowth) that can be linked to different reuse applications for the reclaimed water.
• Such a systematic approach to set sensor targets could be used in the development of WRFs that aim to cover a range of reuse applications with different risks to human health.

## CRediT authorship contribution statement

**Eva Reynaert:** Conceptualization, Methodology, Investigation, Formal analysis, Writing – original draft, Writing – review & editing, Visualization. **Flavia Gretener:** Investigation, Formal analysis, Writing – review & editing. **Timothy R. Julian:** Conceptualization, Writing – review & editing. **Eberhard Morgenroth:** Conceptualization, Writing – original draft, Writing – review & editing.

## Declaration of Competing Interest

The authors declare that they have no known competing financial interests or personal relationships that could have appeared to influence the work reported in this paper.

## Data Availability

All data is available through Eawag's Research Data Institutional Collection.

## Appendix Supplementary materials

Image, application 1

Image, application 2
