# Supplementary material for: Sensor setpoints that ensure compliance with microbial water quality targets for membrane bioreactor and chlorination treatment in on-site water reuse systems
Source: Water Res X. 2022 Dec 27;18:100164. doi: 10.1016/j.wroa.2022.100164 (PMC10214293; doi:10.1016/j.wroa.2022.100164)
Supplement: Supplementary file 1 [file mmc1.docx]

Supplementary information for:

Sensor Setpoints that Ensure Compliance with Microbial Water Quality for Membrane Bioreactor and Chlorination Treatment in On-Site Water Reuse Systems

Eva Reynaert^a,b,*^, Flavia Gretener^a,b^, Timothy R. Julian^a,c,d^, Eberhard Morgenroth^a,b^

^a^ Eawag, Swiss Federal Institute of Aquatic Science and Technology, 8600 Dübendorf, Switzerland Environmental Engineering, 8093 Zürich, Switzerland

^b^ ETH Zürich, Institute of Environmental Engineering, 8093 Zürich, Switzerland

^c^ Swiss Tropical and Public Health Institute, 4051 Basel, Switzerland

^d^ University of Basel, 4055 Basel, Switzerland

^*^ Corresponding author: [eva.reynaert@eawag.ch](mailto:eva.reynaert@eawag.ch)

Submitted to *Water Research X*

# Supplementary Information 1: feed composition

**Source-separated toilet flush water**: The recipe for the source-separated toilet flush water is based on Ziemba et al. (2020), and consisted of 500 g wet feces, 1000 mL urine, and 50 g of soap (see recipe below) mixed with 40 L groundwater. 1 L of concentrated MS2 stock solution was added to the concentrated feed.

The soap consisted of 140 g sodium dodecyl sulphate (SDS), 50 g glycerol and 0.72 g lactic acid, dissolved in tap water for a final volume of 1 L.

**Concentrated handwashing water**: The composition of the concentrated handwashing water is based on Ziemba et al. (2018) and described in Table S.1.

**Table S.1.** Recipe for the concentrated handwashing water.

| Chemical | To be added to 27 L  of tap water [g] |
| --- | --- |
| Sodium dodecyl sulphate (SDS) | 113.4 |
| Humic acid | 1.377 |
| NH_4_NO_3_ | 9.633 |
| NaNO_3_ | 10.23 |
| HNa_2_O_4_P$\cdot$2H_2_O | 7.26 |
| KCl | 0.804 |
| Cl_2_Fe.4H_2_O | 0.75 |
| Glycerol | 40.5 |
| Lactic acid | 0.594 |
| Cl_2_Mn$\cdot$4H_2_O | 0.00319 |
| Cl_2_Co$\cdot$6H_2_O | 0.00045 |

# Supplementary Information 2: disinfection batch tests

**Experimental set-up**: MS2 stock solution (1 mL, previously filtered at 0.45 µm) was spiked into 0.5 L glass bottles containing unchlorinated water from the clean water tank (CWT) to reach an MS2 concentration of 10^8^ plaque forming units (PFU)/mL. Sodium hypochlorite solution was added to reach free chlorine concentrations between 0.05 and 1.75 mg/L at t = 0. Frequent samples were taken for MS2 and the intact cell concentration (ICC) for 30 minutes (batch 1) or 330 min (batch 2, corresponding to the hydraulic residence time in the CWT).

**Results**: Figure S.1 shows a strong tailing effect for both the log-removal values (LRVs) of MS2 and the ICC, with stabilization of the log-removal values (LRVs) within the first few minutes of the experiment. Longer contact times did not further increase the LRV.


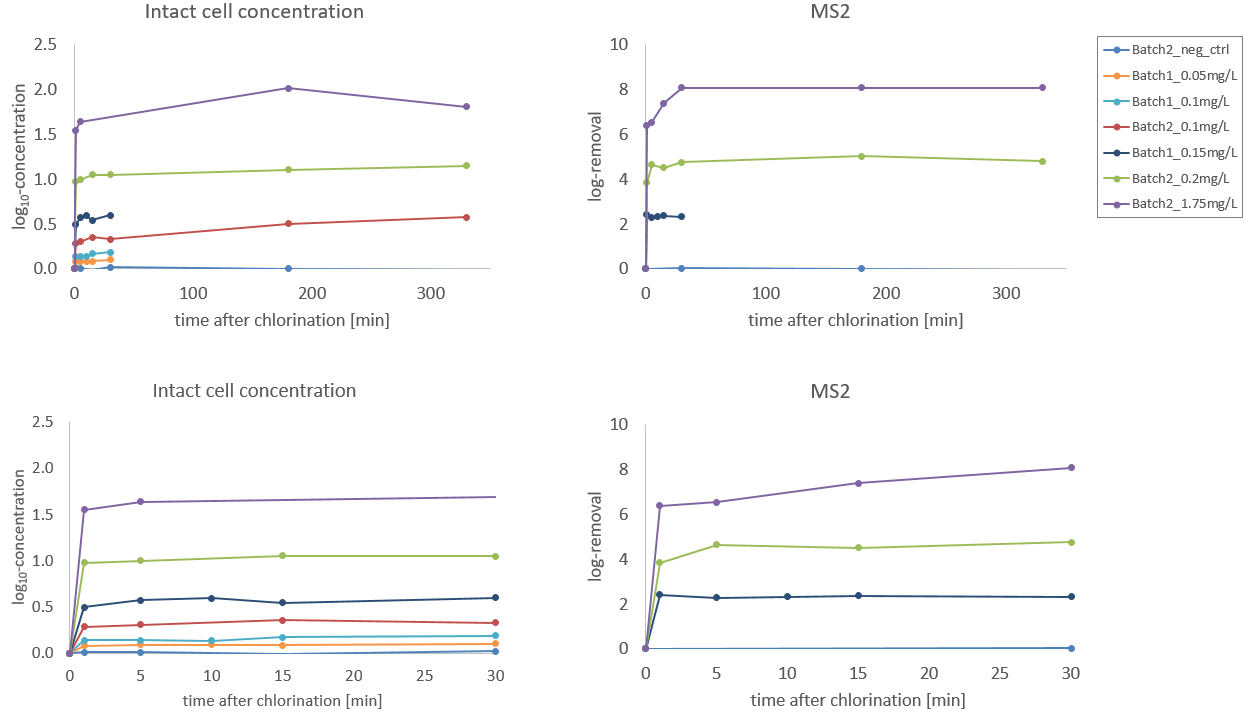


**Figure S.1.** Log-removal values (LRVs) of intact cell concentrations and MS2 over 330 min after chlorination (A and C) and over 30 minutes after chlorination (B and D). 330 min correspond to the hydraulic retention time in the clean water tank. 30 min is the time interval over which chlorine concentrations were stable (exception: 1.75 mg/L chlorine batch, only stable for ~5 min). Sodium hypochlorite solution was added at t = 0. For the 1.75 mg/L chlorine batch, the maximum detectable log-removal was reached after 30 min. Neg_ctrl: negative control (no chlorine added)

# Supplementary Information 3: water quality measured offline

Figure S.2 presents the laboratory results for dissolved organic carbon, ammonium, nitrite and nitrate in the clean water tank (CWT).

| 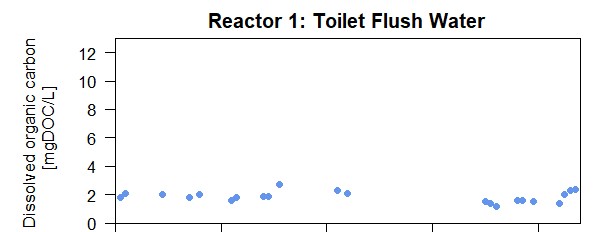 | 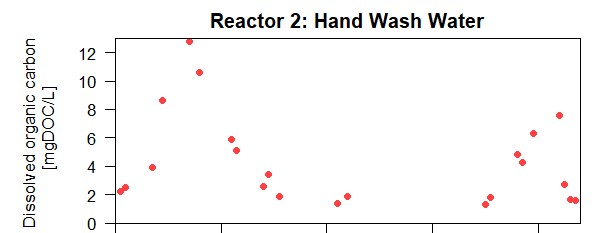 |
| --- | --- |
| 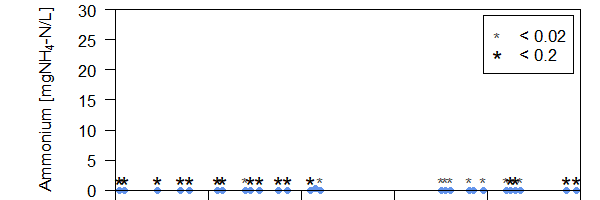 | 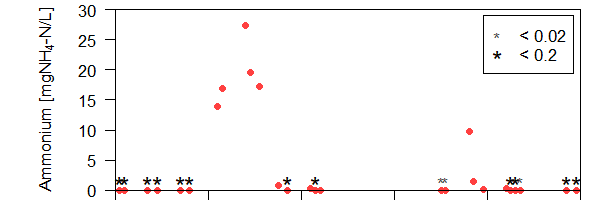 |
| 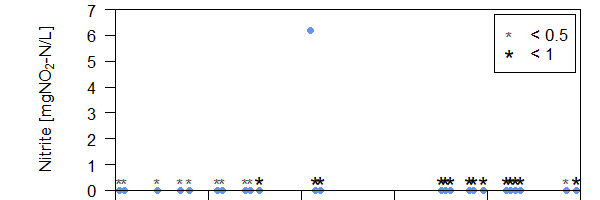 | 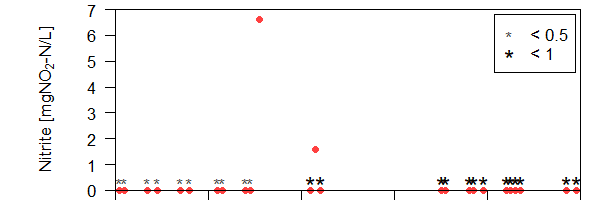 |
| 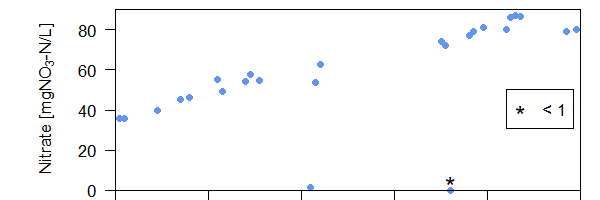 | 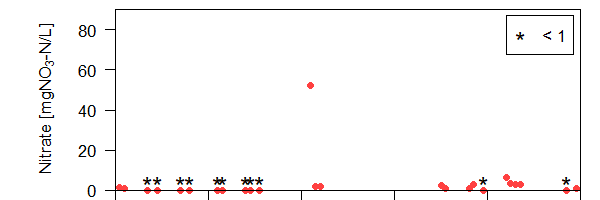 |
| 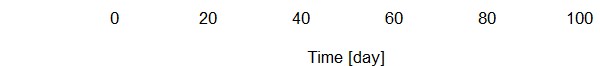 | 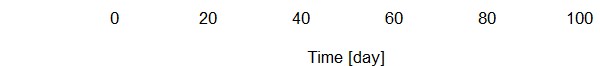 |

**Figure S.2.** Concentrations of dissolved organic carbon, ammonium, nitrite and nitrate in the clean water tank (CWT).

# Supplementary Information 4: cross-correlation plots

Figure S.3 presents the cross-correlation plots for all microbial and sensor data.


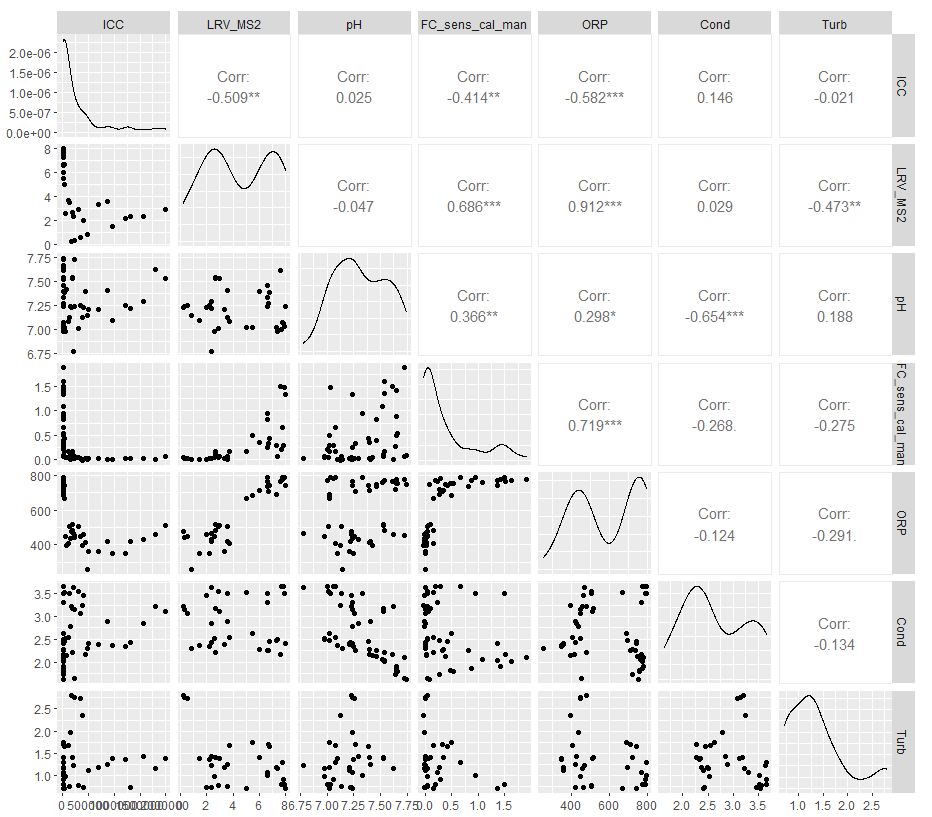


**Figure S.3.** Cross-correlation plots. ICC: intact cell concentration; LRV_MS2: log removal value of MS2; FC_sens_cal_man: calibrated free chlorine concentration; ORP: oxidation-reduction potential; Cond: conductivity; Turb: turbidity.

# Supplementary Information 5: relationship between ${(ORP-{ORP}_{baseline})}^{n_{ORP}}$ and ${(FC-FC_{baseline})}^{n_{FC}}$

If the Chick-Watson models were exact representations of the disinfection processes for both, FC and ORP, then the following equation would be true:

| ${(FC-FC_{baseline})}^{n_{FC}}$ = ${K(ORP-{ORP}_{baseline})}^{n_{ORP}}$ | (S.1) |
| --- | --- |

where ${K= k_{FC}^{'}/k}_{ORP}^{'}$

The best fit model parameters resulted in the same n_FC_ for both microbial indicators (ICC and MS2, n_FC_ = 0.3), however, n_ORP_ was different (0.96 for MS2 and 1.4 for ICC). This means that technically, the relationship from (S.1 cannot be exactly true. Figure S.4 shows the relationships for both n_ORP_, with a relatively good fit for both. It is possible that both values for n_ORP_ would collapse with more data.


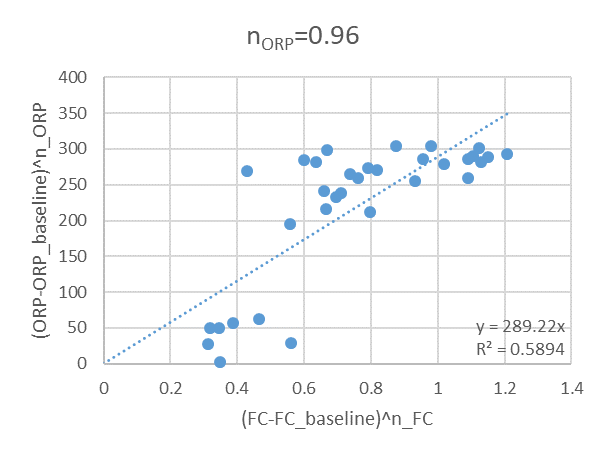

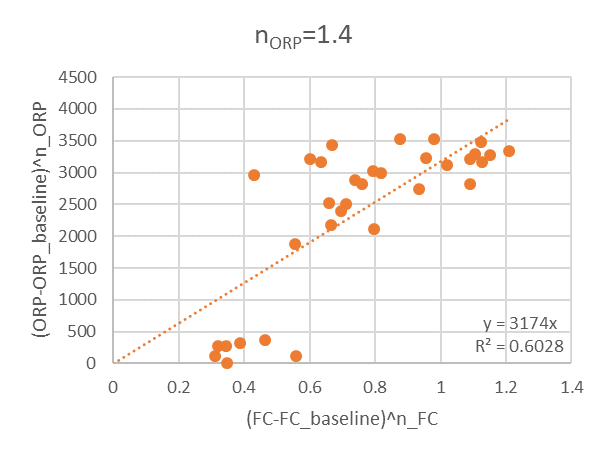


**Figure S.4.** Relationship between ${(ORP-{ORP}_{baseline})}^{n_{ORP}}$ and ${(FC-FC_{baseline})}^{n_{FC}}$_ORP, for n_ORP_ = 0.96 (for MS2) and n_ORP_ = 1.4 (for ICC).

The slopes K are 289.22 (for n_ORP_ = 0.96) and 3174 (for n_ORP_ = 1.4). These should be the same as k’_FC_/k’_ORP_. These relationships are validated in Table S.2.

**Table S.2.** Comparison of slope K (from Figure S.5.) with the ratio between k’_ORP_ and k’_FC_.

| Parameter | Value | k’_FC_/k’_ORP_ | K |
| --- | --- | --- | --- |
| k'_ORP__for MS2 | 0.022 | 268 | 282 |
| k'_FC_  for MS2 | 5.9 |  |  |
| k'_ORP_ for ICC | 0.00076 | 2763 | 3174 |
| k'_FC_ for ICC | 2.1 |  |  |

# Supplementary Information 6: logistic regression using Bayesian analysis

Figure S.5 present the logistic regression models using Bayesian analysis with a weak prior.


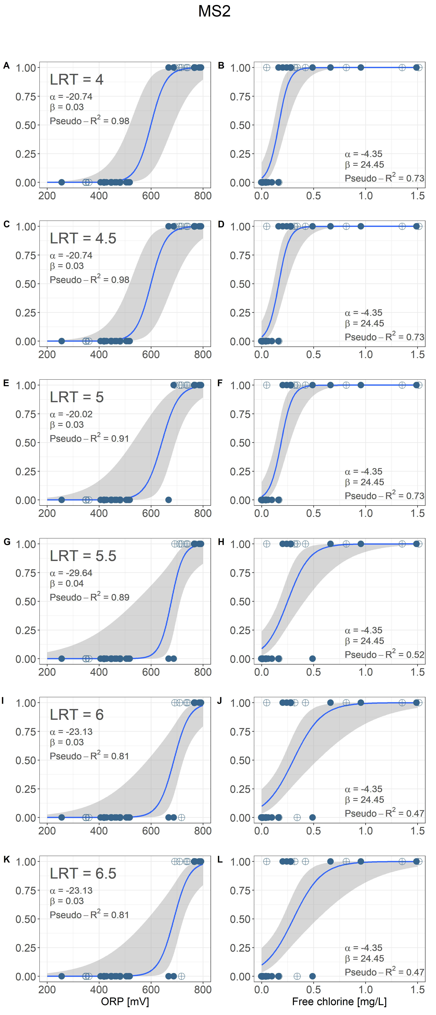

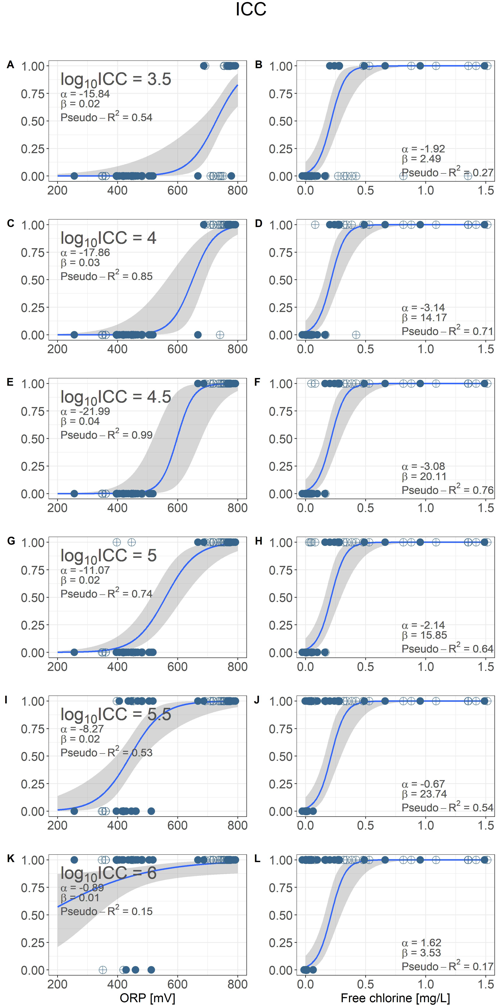


**Figure S.5.** Logistic regression models for log-removal value (LRV) of MS2 and log_10_-value of the intact cell concentration (ICC) as a function of ORP and free chlorine concentrations using Bayesian analysis with an uninformative prior (R package arm, version 112-2). LRT: log-removal target for MS2. 0 = microbial indicator does not meet the microbial water quality target, 1 = meets target. α and β are parameters for the logistic equation, while McFadden’s pseudo-R^2^ evaluates the goodness of fit. The grey areas represent bootstrapped 90% confidence intervals. Blue filled circle: reactor treating handwashing water. Blue crossed circle: reactor treating source-separated toilet flush water.

# Supplementary Information 7: water reuse frameworks

Provided as separate file.

**References**

Ziemba, C., Larivé, O., Reynaert, E., Huisman, T., & Morgenroth, E. (2020). Linking Transformations of Organic Carbon to Post-Treatment Performance in a Biological Water Recycling System. *Science of the total environment, 721*, 137489. doi:<https://doi.org/10.1016/j.scitotenv.2020.137489>

Ziemba, C., Larivé, O., Reynaert, E., & Morgenroth, E. (2018). Chemical Composition, Nutrient-Balancing and Biological Treatment of Hand Washing Greywater. *Water research, 144*, 752-762. doi:<https://doi.org/10.1016/j.watres.2018.07.005>
